# Efficacy and Safety of Vaginal Estradiol Hemihydrate as an Adjunct to Oral Estradiol Valerate in Hormone Replacement Therapy-Frozen Embryo Transfer (HRT-FET) Cycles: A Retrospective Study

**DOI:** 10.7759/cureus.104347

**Published:** 2026-02-26

**Authors:** Jatin Shah, Dharam Shah, Aparna Gangadharan, Vishal Dave, Vivek Sharma

**Affiliations:** 1 Obstetrics and Gynecology, Kamala Polyclinic and Nursing Home, Mumbai, IND; 2 Reproductive Medicine, Kamala Polyclinic and Nursing Home, Mumbai, IND; 3 Medical Affairs, Intas Pharmaceuticals Limited, Ahmedabad, IND

**Keywords:** endometrial thickness, estradiol hemihydrate, estradiol valerate, frozen embryo transfer, hrt-fet, ivf

## Abstract

Objective: To evaluate the efficacy and safety of vaginal estradiol hemihydrate as an adjunct to oral estradiol valerate in improving endometrial thickness and pregnancy outcomes in the hormone replacement therapy-frozen embryo transfer (HRT-FET) cycle.

Materials and methods: This retrospective observational study was conducted at Kamala Polyclinic and Nursing Home, Mumbai, India, and included women aged 25-42 years undergoing HRT-FET treatment cycles between May and July 2024. Only those patients were included in this study whose endometrial thickness was <8 mm after administering oral estradiol valerate 12 mg OD for 12 to 15 days and who achieved a clinically adequate endometrium after co-treatment with vaginal estradiol hemihydrate 2 mg twice daily for two to three days. The study endpoints were measured as an increase in endometrial thickness, clinical pregnancy rate, and safety.

Results: Mean (SD) endometrial thickness increased significantly from 7.47 (0.97) mm to 8.68 (1.34) mm after vaginal supplementation (p < 0.001). The clinical pregnancy rate was 50%. No adverse events were reported.

Conclusion: In this study, administration of estradiol hemihydrate by vaginal route as an adjuvant helped to increase the endometrial thickness and was associated with higher clinical pregnancy in women with a thin endometrium following orally administered estradiol valerate alone.

## Introduction

Globally, infertility was seen at 1.8% in men and 3.7% in women [1]. In the Indian context, about 15 to 20 million couples are suffering from infertility [2]. However, with the advent of in-vitro fertilization (IVF), the scenario has totally changed, and a major chunk of infertile couples can now conceive. In the Indian context, the first IVF baby was born in 1978 in Kolkata (IVF carried out by Dr. Subhas Mukerji) [3]. Recently, in the year 2021, the Assisted Reproductive Technology (ART) Act was passed by the Indian Parliament with the objective of streamlining the ART practice in India in terms of increased transparency and better patient care [4].

As far as embryo transfer (ET) in ART is concerned, conventionally, fresh ET directly after hyperstimulation was carried out. However, with the advancement of technology, the number of thawed frozen embryo transfer (FET) has increased, and this has led to reduced risk of ovarian hyperstimulation syndrome [5]. The optimal endometrial thickness and receptivity can be achieved by various endometrial regimens for FET. They are also required for synchronizing endometrial and embryo development. Hormone replacement therapy (HRT) cycle for FET was originally developed to allow ETs in recipients of donor oocytes [6]. It involves suppression of the natural menstrual cycle and the dominant follicle with the use of exogenous estradiol and sometimes with the addition of a long luteal agonist [7]. Both the natural and synthetic estrogens may be used and can be administered orally, parentally, or vaginally [8].

The receptivity of the endometrium and pregnancy outcomes following ET in the course of IVF/intra-cytoplasmic sperm injection (IVF/ICSI) cycles are indicated by endometrial thickness. Appropriate endometrial thickness is crucial for successful implantation. Also, many studies report low rates of pregnancy in the presence of a thin endometrium [9]. The ideal thickness of the endometrium for conception has varying opinions based on clinical judgement. However, the rate of pregnancy and implantation is negatively correlated with endometrial thickness <7 mm. A thin endometrium also increases the risk of early pregnancy loss [10]. Patients preparing for FET, who have a persistent thin endometrium (<8 mm), need supplementation with additional exogenous estrogen or some other intervention, such as the addition of sildenafil, L-arginine, aspirin, or intrauterine platelet-rich plasma (PRP) [6,11].

However, there is a challenge with oral estradiol tablets as they cannot be given in very high doses, as it is linked to a high risk of thrombosis [12]. To counter this limitation, the vaginal route for administering estradiol tablets has been used in patients with a thin endometrium who have not responded well to orally administered estradiol. Vaginal estradiol may improve the endometrial development not only by influencing the endometrial thickness but also the endometrial microenvironment, while ensuring that the risk of thrombosis is kept minimal due to avoidance of first-pass liver metabolism [13,14]. Therefore, some practitioners do not increase the dose of oral estradiol beyond a particular limit (which may vary among practitioners) and add an alternative route of estradiol administration for endometrial preparation in FET cycles. However, whether these alternative routes are equally efficacious for endometrial preparation is something to be studied. Globally, there are studies comparing oral versus vaginal estradiol administration in terms of efficacy and safety, but similar studies on the Indian population are lacking. Hence, the present study was undertaken to fill this gap in the area of research.

## Materials and methods

Study design

This was a retrospective, investigator-initiated study to evaluate the efficacy and safety of estradiol hemihydrate tablets (used vaginally) as an adjunct to oral estradiol valerate tablets in patients with thin endometrium for endometrial preparation in hormone replacement therapy-frozen embryo transfer (HRT-FET) cycles. All the records of patients registered for HRT-FET cycles at Kamala Polyclinic and Nursing Home, Mumbai, were assessed for eligibility based on inclusion and exclusion criteria.

Inclusion and exclusion criteria

Only patients with top-quality embryos (with self or donor eggs) were included and who underwent artificial endometrial preparation by oral estradiol valerate tablets and subsequently co-administered vaginal estradiol hemihydrate tablets owing to thin endometrium (<8 mm), followed by FET cycles at Kamala Polyclinic and Nursing Home, Mumbai, from May 2024 to July 2024 were included in the study. Patients who were below 25 years of age or above 42 years of age, had a history of smoking, Asherman’s syndrome, malignancy, HIV infection, or tubercular endometritis were excluded.

Study protocol

This was a single-arm retrospective study to evaluate the efficacy and safety of vaginal administration of tablet estradiol hemihydrate (tablet Endofert H 2 mg, marketed by Intas Pharmaceuticals Ltd., Ahmedabad, Gujarat, India) in patients with thin endometrium after administering tablet estradiol valerate orally (tablet Progynova 2 mg) for endometrial preparation in HRT-FET cycles.

On the second day of the menstrual cycle, the HRT regimen was started. Endometrial thickness was measured at the midsagittal plane between the two echogenic endometrial boundaries. All of the women received oral estradiol valerate tablets at a dose of 12 mg per day for 12 to 15 days during the research period (May 2024 to July 2024). If the endometrial thickness was less than 8 mm, tablet estradiol hemihydrate was administered vaginally (2 mg twice daily) for two to three days. Clinical pregnancy was confirmed by the presence of intra-uterine fetal cardiac activity at six to seven weeks of gestation by transvaginal ultrasonography.

Data compilation and analysis

IBM SPSS Statistics for Windows, Version 20 (Released 2011; IBM Corp., Armonk, New York, United States), Student's t-test, and chi-square test were used for the statistical calculations. A p-value < 0.05 was considered significant.

Ethical consideration

The study protocol was reviewed and approved by Indira IVF Hospital Institutional Ethics Committee (Reg. No.: ECR/1629/Inst/MH/2021). The study was conducted in accordance with the ethical principles that have their origin in the Declaration of Helsinki [15] and in accordance with the International Conference on Harmonization’s Good Clinical Practice (ICH-GCP) guidelines [16], New Drugs and Clinical Trial (NDCT) Rules - 2019 [17], ICMR Guidelines for Biomedical Research (2017) [18], applicable regulatory requirements, and in compliance with the protocol. This was a retrospective study without patient identifiers; hence, the informed consent of patients was not taken.

## Results

A total of 50 patients were included in the analysis who met the inclusion criteria, and the median (range) age of the participants was 34.50 (27.00-49.00) years, while the median (range) duration of infertility was 6 (1-15) years. The major causes of infertility included diminished ovarian reserve (DOR) (58%), polycystic ovary syndrome (PCOS) (22%), male factor infertility (16%), uterine factors (10%), bad obstetric history (BOH) (10%), unexplained infertility (10%), and tubal disease (8%), as shown in Table 1.

**Table 1:** Baseline demographic characteristics * Age data were available for 48 patients; ** Duration of infertility data were available for 32 patients; *** Serum AMH value was available for 42 patients. IVF: in-vitro fertilization; PCOS: polycystic ovary syndrome; AMH: anti-Mullerian hormone; POF: prematue ovarian failure; BOH: bad obstetric history; DOR: diminished ovarian reserve; OAT: oligoasthenoteratozoospermia; IUI: intra-uterine insemination

Characteristics	N = 50
Age (years), median (range)*	34.50 (27.00-49.00)
Duration of infertility (years), median (range)**	6.00 (1.00-15.00)
Serum AMH level (ng/mL), median (range)***	1.39 (1.00-17.00)
Indications for IVF, n (%)	
Tubal disease	4 (8)
Endometriosis	1 (2)
Unexplained	5 (10)
DOR	29 (58)
PCOS	11 (22)
Male factor	8 (16)
Other causes, n (%)	
POF	1 (2)
Uterine factor	5 (10)
BOH	5 (10)
Advanced age	4 (8)
OAT	1 (2)
Failed IUI	2 (4)
Hypogonadotropic hypogonadism	1 (2)
Previous ectopic pregnancy	1 (2)
Failed IVF cycle	1 (2)
Baby expired	1 (2)

The median duration of vaginal usage of the estradiol hemihydrate tablet was 2.50 days (Table 2). There was a significant increase in mean (SD) endometrial thickness from 7.47 (0.97) mm to 8.68 (1.34) mm after administering vaginal estradiol hemihydrate (Student's t-test) (p < 0.001). The clinical pregnancy rate (CPR) of the patients included in this study was 50% (22/44 patients; the data of six patients were not available). No major side effects were observed due to vaginal estradiol therapy in any patient.

**Table 2:** Details of vaginal usage of estradiol hemihydrate tablet and outcome (N = 50)

Vaginal estradiol hemihydrate usage details	Mean (SD), median (range)
Days of vaginal estradiol hemihydrate tablet	2.56 (0.61), 2.50 (2.00-4.00)
Endometrial thickness before administering vaginal estradiol hemihydrate tablet (mm)	7.47 (0.97), 7.75 (5.00-9.80)
Endometrial thickness after administering vaginal estradiol hemihydrate (mm)	8.68(1.34), 8.70 (5.00-11.80)

## Discussion

Thin endometrium is well established as one of the common causes of an IVF-ET failure. For successful implantation, the adequate endometrial thickness should be within the range of 6-8 mm when measured in the late proliferative phase [19]. Ultrasound-assisted endometrial thickness measurement is considered a reproducible and simple method for evaluating endometrial growth. Endometrial thicknesses of <7 mm and >14 mm were associated with low pregnancy rates. It has been shown by Liu et al. that between 1.5% and 9.1% of women on assisted reproductive technology (ART) had a thin endometrium [20].

The prolonged estradiol exposure or adjuvant treatments such as low-dose aspirin, vaginal administration of sildenafil citrate, pentoxifylline, vitamin E, and tamoxifen are a few of the strategies commonly used to increase endometrial thickness in individuals with an unresponsive endometrium [21].

While estradiol priming for endometrial preparation during FET cycles is one of the most commonly used protocols, challenges with the oral estradiol therapy in terms of thromboembolism pose a dilemma for clinicians. The risk of veno-thromboembolism (VTE) in oral versus non-oral intake of HRT is 1.8 times higher (odds ratio (OR) 1.8, 95% confidence interval (CI) 1.35-2.29). Hormonal therapy is linked to thrombotic risk mainly by estrogen dose and oral administration, although the mechanisms are not fully understood. It has been demonstrated that estradiol increases both thrombin production and the amount of D-dimer. Furthermore, hormone treatment contributes to the control of endothelial function. According to some accounts, estrogens play a dose-dependent function in the development of matrix metalloproteinases, which break down the collagen and elastin in the intima, causing venous stasis, increased vascular permeability, and ultimately venous thrombosis [12].

Alternate routes of administration of estrogen, in terms of the vaginal route and transdermal route, have been used to circumvent this challenge [8]. There is still debate over the best way to prepare the endometrium for the FET [22].

Vaginal estradiol can be a useful substitute, particularly for individuals who have a poor endometrial response to orally administered estradiol. Of course, as a first-line treatment, oral estradiol pills are more convenient and economical. When given vaginally, estradiol is preferentially absorbed by the endometrium, and at the same time, enough is absorbed into the bloodstream. This appears to be a very effective way to deliver estradiol directly to the targeted tissue, as seen by the high levels of serum estradiol that are acquired, roughly 10 times greater than those obtained after oral administration, and the much higher levels seen in endometrial tissue. Hepatic globulin and lipoprotein production is similar via both oral and vaginal routes, despite 10-fold higher serum estradiol levels after vaginal administration. Therefore, the intravaginal route has a better safety profile in terms of thrombo-embolic disorders [23-27].

The goal of the current study was to assess the vaginal route's effectiveness and safety in an Indian setting. In the present study, vaginal estradiol hemihydrate was administered (2 mg twice daily) to all patients for two to three days, where the endometrial thickness was less than 8 mm after giving oral estradiol valerate 12 mg/day for 12 to 15 days. Vaginal estradiol hemihydrate was administered for a median (range) duration of 2.5 (2.0-4.0) days. There was a significant increase in mean (SD) endometrial thickness from 7.47 (0.97) mm to 8.68 (1.34) mm after administering vaginal estradiol hemihydrate (p < 0.001).

In a similar study (retrospective single center cohort study) conducted by Liao et al., patients who were switched from oral estradiol to vaginal estradiol after receiving oral estradiol for 12 days for endometrial preparation in FET cycles had a significant increase in endometrial thickness (1.9 ± 1.1 mm (mean ± SD)) as compared to the group who were continued on oral estradiol (1.5 ± 0.9 mm (mean±SD)) (p < 0.01). The implantation rate (50.8% vs. 46.1%) and CPR (66.9% vs. 58.0%) were higher in the vaginal estradiol group as compared to the oral estradiol group, though the results were not statistically significant [28].

In another study conducted by Fanchin et al., vaginal versus oral estradiol administration was compared in 39 infertile women in a crossover design. On day 14, the endometrial thickness was higher in the vaginal estradiol group as compared to the oral estradiol group (8.7 ± 0.6 mm vs. 7.1 ± 0.3 mm; p < 0.0001) [25]. Based upon the results of Liao et al., Fanchin et al., and our study, it can be implied that vaginal estradiol was efficacious in terms of improving the endometrial thickness in resistant or less responsive cases to oral estradiol therapy.

To evaluate the clinical impact of the estradiol therapy for endometrial preparation in FET cycles via different routes (oral versus vaginal), Davar et al. conducted a study in 79 women who had undergone an FET cycle. The early miscarriage rate in the vaginal estradiol group was significantly lower than the oral estradiol group (2.5% vs. 18.8%; p = 0.04). On comparing the CPR per transfer, it was found that the vaginal estradiol group had a higher CPR than the oral estradiol group (22.5% vs. 15.6%), though the results were not statistically significant [19].

Thus, based upon our results, it can be suggested that the vaginal route is an effective way to deliver estradiol to the endometrium and is comparable to oral administration. This, perhaps, is a result of enhanced endometrial thickness and adequate endometrial receptivity created by vaginal estradiol therapy that ultimately helps in better embryo implantation [27].

Limitations

This study is limited by its retrospective nature and absence of a control arm. To further clarify the therapeutic advantages of vaginal estradiol administration for endometrial preparation in HRT-FET cycles, prospective randomized clinical studies are required. Confounding factors such as embryo quality could impact the results.

## Conclusions

In this study, administration of estradiol hemihydrate by vaginal route as an adjuvant helped to increase the endometrial thickness and was associated with higher clinical pregnancy in women with a thin endometrium following orally administered estradiol valerate alone.

## References

[1] Liang Y, Huang J, Zhao Q (2025). Global, regional, and national prevalence and trends of infertility among individuals of reproductive age (15-49 years) from 1990 to 2021, with projections to 2040. Hum Reprod.

[2] Katole A, Saoji AV (2019). Prevalence of primary infertility and its associated risk factors in urban population of central India: a community-based cross-sectional study. Indian J Community Med.

[3] Bharadwaj A (2016). The Indian IVF saga: a contested history. Reprod Biomed Soc Online.

[4] Kashyap S, Tripathi P (2024). Assisted Reproductive Technology (Regulation) Act 2021: critique and contestations. Asian Bioeth Rev.

[5] Tocariu R, Niculae LE, Niculae AȘ, Carp-Velișcu A, Brătilă E (2024). Fresh versus frozen embryo transfer in in vitro fertilization/intracytoplasmic sperm injection cycles: a systematic review and meta-analysis of neonatal outcomes. Medicina (Kaunas).

[6] Mumusoglu S, Polat M, Ozbek IY (2021). Preparation of the endometrium for frozen embryo transfer: a systematic review. Front Endocrinol (Lausanne).

[7] Glujovsky D, Pesce R, Sueldo C, Quinteiro Retamar AM, Hart RJ, Ciapponi A (2020). Endometrial preparation for women undergoing embryo transfer with frozen embryos or embryos derived from donor oocytes. Cochrane Database Syst Rev.

[8] Mackens S, Santos-Ribeiro S, van de Vijver A, Racca A, Van Landuyt L, Tournaye H, Blockeel C (2017). Frozen embryo transfer: a review on the optimal endometrial preparation and timing. Hum Reprod.

[9] Mathyk B, Schwartz A, DeCherney A, Ata B (2023). A critical appraisal of studies on endometrial thickness and embryo transfer outcome. Reprod Biomed Online.

[10] Al-Ghamdi A, Coskun S, Al-Hassan S, Al-Rejjal R, Awartani K (2008). The correlation between endometrial thickness and outcome of in vitro fertilization and embryo transfer (IVF-ET) outcome. Reprod Biol Endocrinol.

[11] Lee HJ, Joo JK (2020). When is the optimal timing of frozen embryo transfer after controlled ovarian stimulation?. Ann Transl Med.

[12] Pastori D, Cormaci VM, Marucci S (2023). A comprehensive review of risk factors for venous thromboembolism: from epidemiology to pathophysiology. Int J Mol Sci.

[13] Zhang WY, McCracken M, Dominguez LV, Zhang A, Johal J, Aghajanova L (2024). The impact of estradiol supplementation on endometrial thickness and intrauterine insemination outcomes. Reprod Biol.

[14] Bhupathiraju SN, Grodstein F, Stampfer MJ, Willett WC, Crandall CJ, Shifren JL, Manson JE (2018). Vaginal estrogen use and chronic disease risk in the Nurses' Health Study. Menopause.

[15] (2026). Declaration of Helsinki. https://www.wma.net/what-we-do/medical-ethics/declaration-of-helsinki/.

[16] (2026). ICH harmonised guidelines for good clinical practice E6(R3). https://database.ich.org/sites/default/files/ICH_E6%28R3%29_Step4_FinalGuideline_2025_0106.pdf.

[17] (2026). New Drugs and Clinical Trials Rules. https://www.cdsco.gov.in/opencms/opencms/en/Acts-and-rules/New-Drugs/.

[18] (2026). National Ethical Guidelines for Biomedical and Health Research Involving Human Participants, 2017. https://www.icmr.gov.in/icmrobject/custom_data/pdf/resource-guidelines/ICMR_Ethical_Guidelines_2017.pdf.

[19] Simon A, Laufer N (2012). Repeated implantation failure: clinical approach. Fertil Steril.

[20] Liu KE, Hartman M, Hartman A (2019). Management of thin endometrium in assisted reproduction: a clinical practice guideline from the Canadian Fertility and Andrology Society. Reprod Biomed Online.

[21] Zaha IA, Huniadi A, Bodog F (2023). Autologous platelet-rich plasma (PRP) in infertility-infusion versus injectable PRP. J Pers Med.

[22] Young SL (2013). Oestrogen and progesterone action on endometrium: a translational approach to understanding endometrial receptivity. Reprod Biomed Online.

[23] Davar R, Hoseini M, Saeed L (2020). Vaginal compared oral administration of estradiol in women with thin endometrium: a cross-sectional study. Int J Reprod Biomed.

[24] Gardner DK, Weissman A, Howles CM (2018). Textbook of Assisted Reproductive Techniques. International Journal of Reproductive BioMedicine (IJRM).

[25] Fanchin R, Righini C, Schönauer LM, Olivennes F, Filho JSC, Frydman R (2001). Vaginal versus oral E(2) administration: effects on endometrial thickness, uterine perfusion, and contractility. Fertil Steril.

[26] Tourgeman DE, Gentzchein E, Stanczyk FZ, Paulson RJ (1999). Serum and tissue hormone levels of vaginally and orally administered estradiol. Am J Obstet Gynecol.

[27] Tourgeman DE, Slater CC, Stanczyk FZ, Paulson RJ (2001). Endocrine and clinical effects of micronized estradiol administered vaginally or orally. Fertil Steril.

[28] Liao X, Li Z, Dong X, Zhang H (2014). Comparison between oral and vaginal estrogen usage in inadequate endometrial patients for frozen-thawed blastocysts transfer. Int J Clin Exp Pathol.

